# Depression involved in self-reported prospective memory problems in survivors of breast cancer who have received chemotherapy

**DOI:** 10.1097/MD.0000000000015301

**Published:** 2019-04-19

**Authors:** Zhonglian Huang, Jingjing Zhao, Ke Ding, Yue Lv, Congjun Zhang, Herta H. Chao, Chiang-Shan Li, Huaidong Cheng

**Affiliations:** a Department of Oncology, The Second Affiliated Hospital of Anhui Medical University; b Department of Oncology, The First Affiliated Hospital of Anhui Medical University, Hefei, Anhui; c Cancer Center, VA Connecticut Healthcare System, West Haven; d Department of Internal Medicine, Yale University School of Medicine; e Department of Psychiatry, Yale University School of Medicine, New Haven, CT.

**Keywords:** breast cancer, chemotherapy, depression, prospective memory

## Abstract

To investigate the relationship between depression and the self-reported prospective memory (SPM) problems in breast cancer survivors who have received chemotherapy.

Sixty-three breast cancer patients were administered with self-rating depression scale (SDS) and the prospective memory questionnaire as part of extensive neuropsychological assessments before and after chemotherapy. The performance of SDS and SPM were compared, with the level of significance set at *P* < .05.

Compared with the group before chemotherapy, there is a significant difference on the SPM score (*t* = 6.069, *P* = .000) in breast cancer patients after chemotherapy. Further, there is also a significant difference on the SPM score (*t* = −4.348, *P* = .000) between the patients with and without depression group after chemotherapy.

The present result indicated that the depression in breast cancer survivors after chemotherapy may be involved in their chemotherapy-induced SPM impairment.

## 1 Introduction

Breast cancer is the most common cancer in women.^[1]^ With the development of all kinds of cancer treatment, mortality rates of breast cancer patients had declined in recent years, but the side effects of chemotherapy are reported frequently. Chemotherapy-induced cognitive impairment (CICI) is the most frequent complication reported by breast cancer patients. CICI refers to the impairment of cognitive functions such as memory, attention, and information processing speed that occurs in cancer patients during or after chemotherapy.^[2]^ Recently study suggested that most of breast cancer patients may experience CICI,^[3]^ especially on all kinds of subjective and objective memory impairment.^[4,5]^ The impact of CICI in long-term breast cancer survivor was even worse than the recurrence and metastasis of breast cancer itself. Recently, Wirkner et al^[6]^ found that the levels of cognitive impairment in breast cancer patients were related to their anxiety level. Findings suggested that patients who scored lower on neuropsychological tests reported more symptoms of anxiety.

Recently studies have also suggested that the memory problem was prominent in breast cancer survivors after chemotherapy, and there is some heterogeneity on different memory components.^[3]^ Prospective memory (PM) is the memory required for future plans or intentions and the memory component most closely related to human daily lives. Cheng et al^[7]^ found that breast cancer survivors exhibited PM impairment after chemotherapy. Paquet et al^[8]^ found that chemotherapy-induced PM impairment in breast cancer survivors was associated with symptoms of anxiety and depression.

There is also increasing evidence that subjective memory decline, even with normal performance on objective neuropsychological tests, is associated with an increased risk for developing cognitive decline. Complaints of memory dysfunction are commonly reported by breast cancer survivors after chemotherapy. Our previous study suggested that there was self-reported prospective memory (SPM) impairment in breast cancer survivors after chemotherapy.^[9]^ However, the specific impacting factors are not clear yet. Previous studies have found that emotion is an important factor to affect memory,^[10]^ and depression was one of the most common mental disorders in breast cancer patients,^[11]^ and it may affect their cognitive functions.^[12]^ But up to now, there is no report about whether the depression is related to chemotherapy-induced SPM impairment.

The objective of the present research is to identify whether the depression in breast cancer survivors after chemotherapy is involved in their chemotherapy-induced SPM impairment.

## 2 Methods

### 2.1 Participants

This is a case-control study in breast cancer patients. A total of 63 breast cancer patients who were hospitalized from October 2013 to August 2017 in the Department of Oncology, the Second Affiliated Hospital of Anhui Medical University, were recruited. There were 59 breast cancer patients were identified as those of invasive ductal carcinoma and 4 breast patients as invasive lobular carcinoma by postoperative pathology. All breast patients were right-handed and were selected according to the following criteria:

(1) postoperative pathologic diagnosis of breast cancer;
(2) chemotherapy with paclitaxel and doxorubicin;
(3) mini-mental state examination (MMSE) score ≥24;
(4) Karnofsky performance status score ≥80;
(5) without impairment of vision or hearing and language.

Patients with breast cancer were excluded if the following conditions were present:

(1) patients who have received other treatment such as radiotherapy or endocrine therapy before chemotherapy;
(2) taking relevant drugs to affect cognitive function;
(3) severe diseases of heart, liver, kidney, brain, and the hematopoietic system.

All subjects in the present study were investigated during 1 week before and after chemotherapy. The study was approved by the Research Ethics Committee of the Affiliated Second Hospital of Anhui Medical University, and all subjects provided their informed consent.

### 2.2 Depression assessment

Depression was assessed by self-rating depression scale (SDS), which is a self-rating scale to measure the severity of depression. The degrees of depression were divided into 3 different level: 53 to 62 points, mild depression; 63 to 72 points, moderate depression; and >72 points, severe depression.

### 2.3 Neuropsychological background tests

The MMSE was administered to assess the cognitive functions, including temporal and spatial orientation, short-term memory, calculation, language, and visuospatial skills. The verbal fluency test (VFT) was administered to the subjects, who were instructed to name as many animals as possible in 1 minute. The digit span test (DS) was used to measure short-term memory, in which the subjects were instructed to recall a series of numbers after hearing them in a randomized order.

### 2.4 PM Questionnaires

PM questionnaire (PMQ) were performed individually for each patient before and after chemotherapy. The PMQ is used to test the performance of SPM, and it consists of 8 items for testing PM disorders. The patients were required to rate the degree of their memory failure on a 4-point, Likert scale for each item: (4: very often, 3: sometimes, 2: rarely, 1: never). The total score for PM ranged from 8 to 32, with higher scores indicating greater PM impairment.

### 2.5 Statistical analysis

All data were expressed as mean ± standard deviation. Statistical analysis was performed with SPSS 17.0 software. Scores between before and after chemotherapy were analyzed by means of paired-samples *t* tests. Scores between the depression and nondepression groups were compared using 2 independent samples *t* tests. All statistical tests were 2-tailed, with the level of significance set at *P* < .05.

## 3 Results

### 3.1 The basic clinical information in breast cancer patients

Sixty-three BC patients were evaluated after chemotherapy, out of which, 29 patients were found to have depression, while 34 patients were not. There was no significant difference in age (50.66 ± 7.76 vs 47.35 ± 8.56; *t* = 1.593, *P* = .116) and years of education (7.90 ± 3.36 vs 9.44 ± 3.57; *t* = −1.758, *P* = .084), and other general characteristics between the depression and nondepression groups.

### 3.2 The performance of SDS, MMSE, VFT, DS, and SPM in breast cancer patients before and after chemotherapy

There were a statistically significant difference on SDS (*t* = −5.860, *P* = .000), MMSE scores (*t* = 5.292, *P* = .000) and SPM score (*t* = 6.069, *P* = .000), while no statistically significant differences on VFT and DS in breast cancer patients between before and after chemotherapy group (Table 1).

**Table 1:**
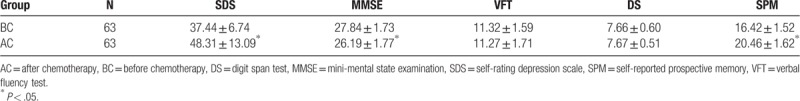
Comparison of performance of SDS, MMSE, VFT, DS, SPM before and after chemotherapy.

### 3.3 Performance of neuropsychological tests and SPM between depression and no depression groups

There were significantly lower on MMSE (*t* = −5.202, *P* = .000) and VFT scores (*t* = −2.591, *P* = .012) of the depression group when compared to nondepression group. Further, as compared with the depression group, the SPM score of the nondepression group was significantly different (*t* = −4.348, *P* = .000) (Table 2).

**Table 2:**
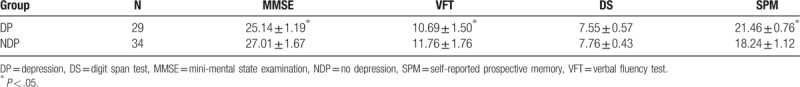
Comparison performance of depression, MMSE, VFR, DS, SPM, between the depression group and the nondepression group after chemotherapy.

## 4 Discussion

As we know, the present research is the first time to evaluate the relationship between depression and the SPM problems in breast cancer survivors after chemotherapy. Previous evidence^[13–15]^ suggested that CICI in breast cancer survivors were mainly related to attention, information processing speed, executive function, language function, visuospatial function, and memory. The present result suggests that depression may contribute to the SPM impairment in breast cancer survivors after chemotherapy.

Memory is one of the important cognitive functions for human, which could be divided into retrospective memory (RM) and PM,^[16]^ and different types of memory involve different region of the brain.^[17]^ Complaints of memory decline are perhaps the most frequently reported cognitive difficulties in breast cancer survivors, which historically has been the exclusive focus of objective memory. PM is one of the most important forms of human memory. PM complaints were significantly more frequent than RM complaints, and were more frequent on self-perceptions regarding day-to-day memory functioning, which required self-initiated intention monitoring, maintenance, and retrieval strategies. Volle et al^[18]^ found that the spontaneous extraction of PM information was related to the functions of the prefrontal cortex. Our previous studies found that there was significant PM impairment in patients with frontal lobe impairment.^[19]^ The present result indicated that breast cancer survivors with depression had significant SPM impairment following chemotherapy, which might be related to insufficient function in the prefrontal structures.

Previous studies investigated the relationship between psychological variable and CICI in breast cancer survivors.^[20,21]^ Depression is a common mental state in breast cancer survivors, and affective distress appears to be a primary contributor to their PM impairment. Higher self-reported levels of depression were significantly associated with increased frequency of SPM complaints. Regression analyses revealed that depression was the predictor of memory complaints.^[22]^ The incidence of depressive symptoms in patients with newly diagnosed breast cancer was up to 32.1%, and in 19.4% of them the depression was moderate or mild, while in 12.7% patients it was moderate or severe.^[23]^

Chemotherapy is one of the important treatment for breast cancer patients; however, most breast cancer survivors worried about chemotherapy. Additionally, the incidence of depression in breast cancer survivors has been reported to be even higher than patients before chemotherapy. Studies have also found that not all breast cancer survivors suffer from depression, and that those with depression have also exhibited differences in the manifestation, degree, and duration of depression.^[22,23]^ Lekovich et al^[24]^ found that the incidence of depression in 95 breast cancer survivors within 1 to 6 months after chemotherapy, and reported it to be up to 67%. Recently studies have found that depression in breast cancer survivors was not temporary, and some patients have even exhibited high levels of depressive symptoms after the treatment.^[25,26]^

The main causes of depression in breast cancer survivors have been reported as follows: shock from the breast cancer diagnosis; special importance of breasts to females, and as most breast cancer patients prefer surgical treatment, the physical change would result in a double whammy physically and mentally; socio-demographic factors such as age, educational level, employment status, household income, obesity, and marital status (all these have been reported to affect patient's depression); the side effects of chemotherapy on fertility, sexual function, perimenopausal period, and related health problems, which have been reported to cause significant anxiety and pain in breast cancer survivors.^[27]^

The present result found that depression caused by various factors might be involved in the occurrence of CICI in breast cancer survivors, especially on SPM impairment. The mechanisms of CICI in breast cancer survivors are not clear yet. However, recently there has been active research in this field. Several studies have found that CICI is manifested differently in breast cancer survivors, with many impacting factors and complex pathogeneses. Therefore, several multidisciplinary studies, such as those on mechanisms of CICI in breast cancer survivors studies have been conducted.^[28,29,30]^ The present result provided evidence for the incidence of SPM impairment in breast cancer survivors after chemotherapy from the perspective of the effect of depression on memory.

## 5 Conclusion

To summarize, the present result suggested that there was a significant decline on general cognitive function and SPM in the breast cancer survivors with depression after chemotherapy. It indicated that depression might be associated with chemotherapy-induced SPM impairment in breast cancer survivors.

## Author contributions

**Conceptualization:** Huaidong Cheng, Jingjing Zhao.

**Data curation:** Zhonglian Huang, Jingjing Zhao, Ke Ding.

**Formal analysis:** Huaidong Cheng, Congjun Zhang, Chiang Shan Li.

**Funding acquisition:** Huaidong Cheng, Yue Lv.

**Investigation:** Jingjing Zhao, Ke Ding, Yue Lv, Huaidong Cheng.

**Methodology:** Huaidong Cheng, Jingjing Zhao, Ke Ding, Herta H. Chao.

**Project administration:** Huaidong Cheng, Congjun Zhang.

**Supervision:** Huaidong Cheng, Congjun Zhang, Herta H. Chao, Chiang Shan Li.

**Writing – original draft:** Zhonglian Huang, Jingjing Zhao, Ke Ding, Yue Lv.
